# Adipokines, hormones related to body composition, and insulin resistance in HIV fat redistribution syndrome

**DOI:** 10.1186/1471-2334-14-347

**Published:** 2014-06-23

**Authors:** Paula Freitas, Davide Carvalho, Ana Cristina Santos, António José Madureira, Esteban Martinez, Jorge Pereira, António Sarmento, José Luís Medina

**Affiliations:** 1 Endocrinology Department, Hospital de São João and University of Porto Medical School, Alameda Hernâni Monteiro, 4200 Porto, Portugal; 2 Department of Clinical Epidemiology, Predictive Medicine and Public Health, University of Porto Medical School, Porto, Portugal; 3 University of Porto Institute of Public Health, Porto, Portugal; 4 Radiology Department, Hospital de São João and University of Porto Medical School, Porto, Portugal; 5 Department of Infectious Diseases, Hospital Clinic, University of Barcelona Medical School, Barcelona, Spain; 6 Nuclear Medicine Department, Hospital de São João, Porto, Portugal; 7 Infectious Diseases Department, Hospital de São João and University of Porto Medical School, Porto, Portugal

**Keywords:** Lipodystrophy, HIV, Adipokines, Body composition, Insulin resistance

## Abstract

**Background:**

Lipodystrophies are characterized by adipose tissue redistribution, insulin resistance (IR) and metabolic complications. Adipokines and hormones related to body composition may play an important role linking these alterations. Our aim was to evaluate adipocyte-derived hormones (adiponectin, leptin, resistin, TNF-α, PAI-1) and ghrelin plasma levels and their relationship with IR in HIV-infected patients according to the presence of lipodystrophy and fat redistribution.

**Methods:**

Anthropometric and metabolic parameters, HOMA-IR, body composition by DXA and CT, and adipokines were evaluated in 217 HIV-infected patients on cART and 74 controls. Fat mass ratio defined lipodystrophy (L-FMR) was defined as the ratio of the percentage of the trunk fat mass to the percentage of the lower limb fat mass by DXA. Patient’s fat redistribution was classified into 4 different groups according the presence or absence of either clinical lipoatrophy or abdominal prominence: no lipodystrophy, isolated central fat accumulation (ICFA), isolated lipoatrophy and mixed forms (MXF). The associations between adipokines levels and anthropometric, metabolic and body composition were estimated by Spearman correlation.

**Results:**

Leptin levels were lower in patients with FMR-L and isolated lipoatrophy, and higher in those with ICFA and MXF. Positive correlations were found between leptin and body fat (total, trunk, leg, arm fat evaluated by DXA, and total, visceral (VAT), subcutaneous adipose tissue (SAT), and VAT/SAT ratio evaluated by CT) regardless of FMR-L, and with HOMA-IR only in patients with FMR-L. Adiponectin correlated negatively with VAT, and its mean levels were lower in patients with ICFA and higher in those with no lipodystrophy. Resistin was not correlated with adipose tissue but positively correlated with HOMA-IR in FMR-L patients. PAI-1 levels were higher in MXF-patients and their levels were positively correlated with VAT in those with FMR-L. Ghrelin was higher in HIV-infected patients than controls despite BMI-matching.

**Conclusion:**

The overall body fat reduction in HIV lipoatrophy was associated with low leptin plasma levels, and visceral fat accumulation was mainly associated with decreased plasma levels of adiponectin.

## Background

Changes in body fat distribution are a common finding in HIV infected patients treated with combined antiretroviral therapy (cART) and this condition has many similarities to rare, congenital and acquired lipodystrophies, which are associated with depletion of subcutaneous fat, increased triglycerides and profound insulin resistance (IR) [1,2]. Adipose tissue traditionally was considered an energy storage organ, but over the last decade, it has emerged as a metabolically active endocrine organ secreting multiple bioactive peptides, collectively called “adipokines”, other proteins including inflammatory mediators such as tumor necrosis factor alpha (TNF-α) and plasminogen activator inhibitor-1 (PAI-1), which not only influence adipocyte function in an autocrine and paracrine fashion, but also affect more than one metabolic pathway through the bloodstream’s systemic circulation [3]. All adipose tissue products seem to contribute significantly in various metabolic processes both at local and systemic levels and may induce proinflammatory state and oxidative damage, leading to initiation and progression of atherosclerosis [4]. On the other hand, these patients experience marked changes in circulating levels of adipocyte secreted hormones, including leptin and adiponectin, contributing to metabolic abnormalities, and hormones related to body composition, such as ghrelin [5]. Ghrelin is an orexigenic gut peptide that is the endogenous ligand for the growth hormone (GH) secretagogue receptor. Low ghrelin may be a marker of metabolic dysregulation and altered body fat without specifically contributing to low GH [6].

cART in HIV infection produces widespread metabolic complications, including hyperlipidemia, IR and lipodystrophy [peripheral fat wasting (lipoatrophy) with or without abdominal/visceral fat accumulation (lipohypertrophy)] [7]. The hormonal changes caused by increased (in lipohypertrophy) or reduced subcutaneous fat (in lipoatrophy) may be central to the metabolic abnormalities observed in HIV-lipodystrophy [6].

The aim of this study was to evaluate the relationship between fat mass, lipodystrophy defined by the Fat Mass Ratio (FMR-L) and the four different categories of fat distribution, and adipocyte derived hormones (adiponectin, leptin, resistin, TNF-α), orexigenic hormones (ghrelin), prothrombotic factors (PAI-1) and IR in HIV-infected patients on cART.

## Methods

### Subjects

As part of a cross-sectional study, 217 consecutive clinically stable HIV-infected adults receiving cART were evaluated between 2005 and 2008 at the Endocrinology Outpatient Department. Seventy four age-, gender- and BMI-matched HIV-uninfected controls were also evaluated. These controls were selected from a previously assembled cohort of non-institutionalized community adults, the EPI-Porto study [8]. This study was approved by the Ethics Committee for Health of Hospital São João and each patient provided written informed consent.

### Clinical assessment

For each patient the following information was collected using a standardized protocol: demographic data (age, gender), duration of HIV infection, HIV infection risk factors, duration of cART and characterization of the infection. We used the “Centers for Disease Control and Prevention” (CDC) criteria for classifying the degree the infection [9]. Weight, height, circumferences of neck, waist, hip, thigh and arm were measured as previously published [10-13]. Blood pressure (BP) was measured using the established recommendations [14].

Clinical lipodystrophy was defined as a peripheral lipoatrophy with or without a central fat accumulation assessed by both patient and practitioner, as previously described [11-13]. Presence of central fat accumulation or abdominal prominence was defined by the measurement of waist circumference using the International Diabetes Federation (IDF) criteria for metabolic syndrome [15]. Patients were classified into four different categories according the presence or absence of either clinical lipoatrophy or abdominal prominence: **no lipodystrophy** - patients without clinical lipoatrophy and without abdominal prominence; **isolated central fat accumulation** - patients without clinical lipoatrophy and with abdominal prominence; **isolated lipoatrophy** - patients with clinical lipoatrophy and without abdominal prominence; **mixed forms of lipodystrophy** - patients with clinical lipoatrophy and with abdominal prominence. The clinical assessment was performed by the same practitioner (PF).

### Evaluation of body composition

Body composition was assessed with whole-body dual-energy X-ray absorptiometry (DXA - Lunar Expert XL). DXA measurement was performed while the patient was in a supine position, with standard positioning of the arms and feet. Markers used in this study for trunk and lower limbs that defined regions of interest as indicated by the manufacturer. Regional fat mass values were grouped and analysed for the following anatomical regions: arms, legs, trunk and total body. The fat mass ratio (FMR) is the ratio of the percentage of the trunk fat mass to the percentage of the lower limb fat mass (FMR = % of the trunk fat mass/% of the lower limb fat mass) [16]. We define lipodystrophy by FMR using the cut-off value of 1.961 for men and 1.329 for women [10]. The quantification of total, visceral, and peripheral abdominal fat was performed with a 64-slice computed tomography (CT) scanner (Siemens Sensation 64 Cardiac) with the same technique as previously described [17,18]. All values were expressed in cm^2^ rounded to the nearest centesimal.

### Laboratory analysis

A venous blood sample was drawn after a 12-hour overnight fast. All the samples were analysed at the central laboratory of our hospital. Patients without a previous diagnosis of diabetes were submitted to a glucose tolerance test (OGTT). The test was performed as described by the World Health Organization using a glucose load containing the equivalent of 75 g anhydrous glucose dissolved in water.

The CD4 cell count was determined by flow cytometry and plasma HIV-1 RNA loads were measured by a quantitative reverse transcriptase polymerase chain reaction (Roche Diagnostic Systems, Inc., Branchburg, NJ, USA), which has a lower limit of detection of 50 copies/mL.

### Adipokines and hormones related to body composition

Leptin (Human Leptin RIA Kit, Linco Research), adiponectin (Human Adiponectin RIA Kit, Linco Research), resistin (Human Resistin ELISA Kit, Linco Research), TNF-α (TNF-α IRMA Kit, BioSource), ghrelin (Ghrelin Active RIA Kit, Linco Research) and PAI-1 (Human PAI-1 ELISA Kit, IBL International GMBH) were measured in the Nobre Laboratory of the Porto Medical School. For leptin, the intra-assay precision is 4.6% and inter-assay precision 5.0%. For adiponectin, the intra-assay precision is 1.8% and inter-assay precision 9.3%. For resistin, the intra-assay precision is 3.2% and inter-assay precision 7.1%. For TNF-α, the intra-assay precision is 3.1% and inter-assay precision 5.7%. For ghrelin, the intra-assay precision is 6.7% and inter-assay precision 9.6%. The overall intra-assay coefficient of variation for PAI-1 was 4.7%.

### Insulin resistance evaluation

Insulin resistance was defined by the homeostasis model assessment of insulin resistance (HOMA) and insulin sensitivity by the quantitative insulin sensitivity check index (QUICKI). These indexes were calculated by the following formulas:

**HOMA-IR index** = (insulin 0 × glucose 0)/22.5 [19].

**QUICKI** = 1/[log (fasting insulin in mU/l) + log (fasting plasma glucose in mg/dL)] [20].

Glucose was expressed in mmol/L and insulin in μUI/mL.

### Statistical analysis

Data were described as mean and standard deviation (SD) for quantitative variables and compared using the Student-t test or the Mann-Whitney test as appropriate. For the comparison between the four groups of fat distribution and adipokines parameters the Kruskall-Wallis test was used. Categorical variables were described as counts and proportions, and compared using the chi-square or Fisher’s exact test.

For estimating the association between adipokines levels and anthropometric, metabolic and body composition, Spearman correlation coefficients were calculated. Statistical analysis was performed using the SPSS version 18.0 software (SPSS Inc., Chicago, Illinois, USA). All probabilities were two tailed and p values <0.05 were regarded as significant, with the exception of the correlations between adipokines levels and anthropometric, metabolic and body composition, considering p values <0.001 as significant after applying the Bonferroni correction.

## Results

### Sample characteristics, adipokines and hormones related to body composition in HIV-infected patients and HIV-uninfected controls

HIV-infected patients had significantly higher waist/hip ratio, glucose, triglyceride, insulin, HOMA, and ghrelin levels, and adiponectin/leptin ratio. However, they had lower weight, hip and arm circumferences, HDL-cholesterol levels, QUICKI, resistin, adiponectin and leptin levels. No differences were observed between HIV-infected patients and HIV-uninfected controls regarding waist circumference, systolic and diastolic blood pressure and total cholesterol, LDL-cholesterol, uric acid, hsCRP and TNF-α levels (Table 1).

**Table 1:** Sample characteristics, adipokines and hormones related to body composition in HIV-uninfected controls and HIV-infected patients

	**HIV-uninfected controls**	**HIV-infected patients**	**p**
n	74	217	
Age [years, mean (sd)]	47.2 (9.7)	46.6 (11.6)	0.73
Gender [n (%)]			
Male	40 (54.1)	144 (66.9)	
Female	34 (45.9)	73 (33.6)	0.08
Weight [Kg, mean (sd)]	71.5 (13.4)	67.8 (12.3)	0.03
BMI [(kg/m^2^), mean (sd)]	26.3 (4.3)	35.1 (4.5)	0.05
Waist circumference [cm, mean (sd)]	90.7 (11.6)	91.2 (11.6)	0.75
Hip circumference [cm, mean (sd)]	99.8. (7.1)	94.5 (8.4)	<0.001
Waist/hip ratio [mean (sd)]	0.91 (0.08)	0.96 (0.08)	<0.001
Arm circumference [cm, mean (sd)]	30.6 (3.5)	27.0 (3.0)	<0.001
Systolic blood pressure [mmHg, mean (sd)]	122.2 (17.3)	121.9 (17.5)	0.99
Diastolic blood pressure [mmHg, mean (sd)]	78.9 (10.1)	76.7 (11.3)	0.24
Glucose [mg/dL, mean (sd)]	91.7 (12.2)	107.7 (41.5)	0.03
Insulin [μU/mL, mean (sd)]	5.5 (4.9)	11.2 (10.6)	<0.001
HOMA [mean (sd)]	1.3 (1.4)	3.1 (3.2)	<0.001
QUICKI [mean (sd)]	0.39 (0.05)	0.36 (0.08)	<0.001
Total cholesterol [mg/dL, mean (sd)]	217.2 (43.6)	224.2 (58.0)	0.26
LDL cholesterol [mg/dL, mean (sd)]	134.9 (38.8)	128.5 (49.9)	0.42
HDL cholesterol [mg/dL, mean (sd)]	57.4 (14.9)	46.1 (13.6)	<0.001
Triglycerides [mg/dL, mean (sd)]	119.8 (57.6)	267.5 (182.8)	<0.001
Uric acid [mg/dL, mean (sd)]	49.6 (15.4)	46.8 (15.0)	0.165
hs-CRP [mg/dL, mean (sd)]	0.36 (0.54)	0.45 (0.73)	0.55
Resistin [ng/mL, mean (sd)]	15.5 (11.0-22.2)	10.4 (8.0-13.9)	<0.001
Adiponectin [μg/mL, mean (sd)]	5.18 (2.80-7.74)	2.68 (1.19-5.46)	<0.001
TNF-α [pg/mL, mean (sd)]	45.3 (29.9-60.5)	44.6 (30.1-73.6)	0.63
Ghrelin [pg/mL, mean (sd)]	18.6 (10.5-25.4)	75.0 (47.7-139.5)	<0.001
Leptin [ng/mL, mean (sd)]	11.8 (6.3-21.3)	3.4 (1.9-7.6)	<0.001
Adiponectin/leptin ratio [mean (sd)]	1.00 (1.95)	2.29 (5.87)	0.02

### Adipokines and hormones related to body composition in HIV-infected patients according to gender

HIV-infected females had higher hip circumference, total fat mass and subcutaneous adipose tissue (SAT) on abdominal CT scan evaluation and total, trunk, leg and arm mass fat by DXA. They also had lower visceral adipose tissue (VAT) and VAT/SAT ratio on CT scan. Also, females had higher leptin levels and lower TNF-α and adiponectin/leptin ratio. No gender-related differences were observed in adiponectin, resistin, ghrelin and PAI-1 levels (Table 2).

**Table 2:** Body composition, adipokines and hormones related to body composition in HIV-infected patients, according to gender

	**Male**	**Female**	**P**
n	144	73	
Age [years, mean (SD)]	46.8 (11.2)	46.2 (12.5)	0.72
BMI [(kg/m^2^), mean (SD)]	24.7 (3.8)	26.1 (5.6)	0.05
Waist circumference [cm, mean (SD)]	91.2 (10.6)	91.2 (13.5)	0.98
Hip circumference [cm, mean (SD)]	93.1 (6.7)	97.2 (10.5)	0.003
Total fat mass CT [cm^3^, mean (SD)]	243.4 (136.2)	356.0 (166.7)	<0.001
VAT CT [cm^3^, mean (SD)]	140.7 (86.2)	113.6 (69.9)	0.02
SAT CT [cm^3^, mean (SD)]	102.6 (76.9)	242.4 (125.7)	<0.001
VAT/SAT ratio	2.2 (2.4)	0.55 (0.40)	<0.001
Total fat mass DXA [%, mean (SD)]	16.8 (8.1)	33.3 (10.0)	<0.001
Trunk fat in DXA [%, mean (SD)]	20.6 (9.8)	33.4 (10.3)	<0.001
Leg fat mass DXA [%, mean (SD)]	12.2 (9.1)	31.9 (12.5)	<0.001
Arm fat mass DXA [%, mean (SD)]	15.2 (8.9)	42.3 (11.4)	<0.001
Adiponectin [μg/mL, median (25th and 75th percentile)]	3.34 (1.79-6.59)	3.00 (1.36-6.55)	0.39
Leptin [ng/mL, median (25th and 75th percentile)]	3.0 (1.8-5.8)	11.2 (5.7-18.0)	<0.001
Resistin [ng/mL, median (25th and 75th percentile)]	11.2 (8.4-17.0)	11.2 (8.9-16.8)	0.42
Ghrelin [pg/mL, median (25th and 75th percentile)]	55.5 (26.9-99.2)	44.0 (21.4-79.2)	0.07
PAI-1 [pg/mL, median (25th and 75th percentile)]	61.3 (38.5-104.3)	43.4 (30.8-95.8)	0.37
TNF-α [pg/mL, median (25th and 75th percentile)]	49.9 (31.6-72.4)	39.7 (27.2-60.0)	0.03
Adiponectin/leptin ratio, median (25th and 75th percentile)	0.945 (0.34-2.04)	0.245 (0.09-0.63)	<0.001

### Adipokines and hormones related to body composition according to the four categories of body composition

Patients with isolated central fat accumulation and mixed forms of lipodystrophy were older and had higher BMI. Patients with no lipodystrophy had higher levels of adiponectin and those with isolated central fat accumulation had the lowest levels of adiponectin. The levels of leptin were higher in patients with isolated central fat accumulation and mixed forms of lipodystrophy and lower in those with isolated lipoatrophy. No significant differences were observed in resistin, ghrelin and TNF-α levels between the four groups of body composition. Patients with no lipodystrophy and mixed forms of lipodystrophy had higher levels of PAI-1, and the lowest levels of PAI-1 were observed in those with isolated central fat accumulation. The adiponectin/leptin ratio was higher in patients with no lipodystrophy and lower in patients with isolated central fat accumulation, followed by the patients with mixed forms of lipodystrophy (Table 3).

**Table 3:** Adipokines and hormones related to body composition according to four categories of body composition

	**No lipodystrophy N = 23**	**Isolated central fat accumulation N = 56**	**Isolated lipoatrophy N = 78**	**Mixed forms of lipodystrophy N = 60**	**P**
Age [years, mean (SD)]	39.9 (11.3)	46.5 (12.6)	44.1 (9.7)	52.6 (10.8)	<0.001
BMI [kg/m^2^, mean (SD)]	23.0 (2.4)	29.1 (4.4)	21.9 (2.8)	26.5 (3.2)	<0.001
Adiponectin [μg/mL, median (25th and 75th percentile)]	4.40 (2.43-8.75)	1.90 (0.89-3.48)	2.64 (1.20-6.53)	2.81 (1.04-5.22)	0.02
Leptin [ng/mL, median (25th and 75th percentile)]	3.0 (2.0-5.7)	10.3 (5.3-15.1)	2.1 (1.4-3.3)	4.3 (2.4-7.0)	<0.001
Resistin [ng/mL, median (25th and 75th percentile)]	9.1 (7.9-12.6)	10.3 (7.2-12.0)	9.5 (8.1-15.3)	11.2 (8.1-16.4)	0.36
Ghrelin [pg/mL, median (25th and 75th percentile)]	72.0 (52.4-110.3)	71.0 (41.3-110.8)	81.9 (46.2-194.3)	79.7 (52.4-122.4)	0.42
PAI-1 [pg/mL, median (25th and 75th percentile)]	111.9 (64.8-162.8)	40.4 (30.6-45.8)	61.3 (28.3-96.4)	68.7 (34.6-109.6)	0.047
TNF-α [pg/mL, median (25th and 75th percentile)]	42.8 (33.1-60.5)	41.6 (27.7-83.4)	49.8 (30.7-72.6)	43.7 (29.6-79.8)	0.91
Adiponectin/leptin ratio median (25th and 75th percentile)	1.30 (0.97-2.50)	0.19 (0.06-0.38)	1.17 (0.43-4.60)	0.73 (0.20-1.43)	<0.001

### Sample characteristics, body composition, metabolic parameters, adipokines and hormones related to body composition, according to the presence lipodystrophy defined by FMR

In this sample of 217 HIV-1 infected patients (144 men and 73 women) on cART, 41% of patients presented lipodystrophy defined by FMR (FMR-L). Table 4 shows the characteristics of the study sample according to the presence of FMR-L. Patients with FMR-L were more frequently male, older and had a longer duration of HIV infection and length of cART. No significant differences were found in weight, BMI, waist, arm and thigh circumferences among patients with or without FMR-L. Patients with FMR-L had a higher neck circumference and waist/hip ratio, but a lower hip circumference. Also, they had significantly a higher mean CD4+ cell count, but no significant difference in viral suppression rate was observed. No differences were observed between patients with and without FMR-L regarding cART regimens and CDC classification. Also, no differences were observed regarding diastolic blood pressure, hs-CRP, total cholesterol, LDL and HDL cholesterol levels. The patients with FMR-L had higher systolic blood pressure, uric acid, triglycerides, HOMA, fasting glucose and insulin levels. Regarding OGTT, glucose and insulin at 2 hours were higher in patients with FMR-L and as expected, QUICKI was lower in patients with FMR-L.

**Table 4:** Sample characteristic, body composition, adipokines and hormones related to body composition according to the presence of lipodystrophy defined by FMR

	**Without lipodystrophy**	**With lipodystrophy**	**P**
n (%)	128 (59.0)	89 (41.0)	-
Gender [n (%)]			
Male	76 (59.4)	68 (76.4)	
Female	52 (40.6)	21 (23.6)	0.01
Age [years, mean (sd)]	44.7 (12.2)	49.4 (10.2)	0.003
Duration of HIV infection [years, mean (sd)]	7.4 (3.9)	8.6 (3.6)	0.007
cART [years, mean (sd)]	5.8 (3.8)	8.0 (3.4)	<0.001
Weight [Kg, mean (sd)]	67.4 (13.0)	68.3 (11.1)	0.60
BMI [(kg/m^2^), mean (sd)]	25.0 (4.8)	25.3 (4.5)	0.68
Waist circumference [cm, mean (sd)]	90.2 (12.2)	92.7 (10.5)	0.10
Hip circumference [cm, mean (sd)]	95.9 (9.2)	92.5 (6.7)	0.002
Thigh circumference [cm, mean (sd)]	48.3 (5.6)	46.9 (4.8)	0.07
Arm circumference [cm, mean (sd)]	26.7 (3.2)	27.2 (2.7)	0.28
Neck circumference [cm, mean (sd)]	36.4 (3.8)	37.9 (3.7)	0.01
Waist/hip circumference ratio	0.94 (0.08)	1.00 (0.07)	<0.001
CD4 cell count [cells/mm^3^, mean (sd)]	498.9 (289.4)	644.6 (354.1)	0.001
HIV RNA (<50) [n (%)]	109 (85.2)	81 (91.0)	0.45
HIV risk factor [n (%)]			
Injecting drug user	36 (28.6)	17 (19.1)	
Homosexual contact	7 (5.6)	16 (18.0)	
Heterosexual contact	81 (64.3)	54 (60.7)	
Others	2 (1.6)	2 (2.2)	0.02
CDC [n (%)]			
A	67 (52.8)	57 (64.0)	0.16
B	2 (1.6)	0 (0)	
C	58 (45.7)	32 (36.0)	
ART [n (%)]			
IP	65 (52.0)	48 (53.9)	0.89
NNRTI	62 (49.6)	45 (50.6)	0.89
NRTI	121 (96.8)	86 (96.6)	1.00
Total Cholesterol [mg/dL, mean (sd)]	218.1 (56.4)	233.1 (59.5)	0.06
LDL-cholesterol [mg/dL, mean (sd)]	125.4 (50.2)	133.0 (49.4)	0.27
HDL- cholesterol [mg/dL, mean (sd)]	47.2 (14.6)	44.4 (11.7)	0.14
Triglycerides [mg/dL, mean (sd)]	244.8 (117.6)	300.1 (195.3)	0.03
Uric acid [mg/L, mean (sd)]	43.8 (14.3)	51.0 (15.1)	<0.001
Systolic blood pressure [mmHg, mean (sd)]	120.0 (18.6)	124.8 (15.6)	0.04
Diastolic blood pressure [mmHg, mean (sd)]	75.7 (11.8)	78.1 (10.5)	0.14
Glucose 0 [mg/dL, mean (sd)]	103.0 (39.5)	114.4 (43.7)	0.002
Glucose 2 h at OGTT [mg/dL, mean (sd)]	123.9 (42.9)	139.7 (49.0)	0.03
Insulin 0 [μU/mL, mean (sd)]	9,96 (10.50)	12.96 (10.55)	0.005
Insulin 2 h at OGTT [μU/mL, mean (sd)]	49. 8 (40.1)	88.3 (142.2)	0.005
HOMA [mean (sd)]	2.65 (3. 01)	3.70 (3.28)	<0.001
QUICKI [mean (sd)]	0.37 (0.10)	0.34 (0.4)	<0.001
hsCRP [mg/dL, mean (sd)]	0.50 (0.90)	0.36 (0.27)	0.28
Adiponectin [μg/mL, mean (sd)]	5.64 (7.39)	3.95 (4.49)	0.23
Leptin [ng/mL, mean (sd)]	7.3 (8.5)	3.9 (3.3)	0.008
Resistin [ng/mL, mean (sd)]	12.8 (13.4)	12.8 (7.4)	0.37
Ghrelin[pg/mL, mean (sd)]	117.4 (133.6)	118.1 (106.7)	0.45
PAI-1 [pg/mL, mean (sd)]	62.6 (43.6)	102.1 (110.1)	0.26
TNF-α [pg/mL, mean (sd)]	61.9 (50.3)	52.3 (33.9)	0.43
Adiponectin/leptin ratio [mean (sd)]	2.72 (7.21)	1.69 (3.13)	0.42
Fat mass [%, mean (sd)] DXA			
Total	24.8 (13.1)	19.3 (8.5)	0.002
Trunk	25.7 (13.3)	24.3 (9.0)	0.53
Leg	24.4 (14.7)	11.1 (7.4)	<0.001
Arm	27.2 (17.1)	20.3 (13.6)	0.001
Fat mass [Kg, mean (sd)] DXA			
Total	17.3 (11.1)	13.2 (7.3)	0.02
Trunk	9.3 (6.0)	9.0 (4.8)	0.69
Leg	5.2 (3.6)	2.1 (1.6)	<0.001
Arm	2.1 (1.9)	1.5 (1.2)	0.008
Body Fat Mass by Quantitative CT			
Total [cm^2^, mean (sd)]	282.9 (174.9)	276.5 (126.6)	0.92
VAT [cm^2^, mean (sd)]	111.2 (85.4)	139.4 (68.7)	<0.001
SAT [cm^2^, mean (sd)]	171.7 (125.9)	117.0 (92.4)	0.001
VAT/SAT ratio [mean (sd)]	1.07 (1.46)	2.44 (2.60)	<0.001

Note: cART - combined antiretroviral therapy; BMI. Body mass index; OGTT- oral glucose tolerance test; DXA - dual-energy X-ray absorptiometry CT - computed tomography; VAT- visceral adipose tissue; SAT - subcutaneous adipose tissue.

On DXA evaluation, patients with FMR-L had lower total, leg and arm fat mass (either in % or Kg) than patients without FMR-L with no significant difference in trunk fat mass. On CT evaluation at abdominal level, patients with FMR-L had higher VAT and lower SAT with a higher central/peripheral fat ratio, with no difference in total fat at this level.

Leptin levels were lower in patients with FMR-L. No significant differences were observed in levels of adiponectin, resistin, ghrelin, PAI-1, TNF-α and adiponectin/leptin ratio among patients with or without FMR-L (Table 4).

### Adipokines, body composition hormones and metabolic parameters

We evaluated the correlations between leptin, resistin, TNF-α and adiponectin with age, waist/hip circumference ratio, arm, waist, hip circumferences, BMI, fasting glucose and insulin, HOMA, triglycerides and total cholesterol in all the HIV-infected patients and HIV-uninfected controls. Leptin was positively associated with arm, waist, hip circumferences, BMI and fasting insulin both in the HIV-infected patients and in HIV-uninfected controls. In HIV-uninfected controls, leptin was also positively correlated with HOMA. A negative correlation was observed between resistin and hip circumference and BMI in the HIV-infected patients, while a positive correlation between resistin and triglycerides was found in the HIV-uninfected controls. Also, a negative correlation was observed between TNF-α and total cholesterol in HIV-infected patients, and between adiponectin and arm circumference in controls (Table 5).

**Table 5:** Associations between adipokines and anthropometric and metabolic parameters in HIV patients and controls

**Correlation between**	**Leptin**				**Resistin**				**TNF-α**				**Adiponectin**			
	**Total of HIV patients**		**Controls**		**Total of HIV patients**		**Controls**		**Total of HIV patients**		**Controls**		**Total of HIV patients**		**Controls**	
	**n = 161**		**n = 67**		**n = 170**		**n = 74**		**n = 160**		**n = 74**		**n = 159**		**n = 74**	
	**r**	**P**	**R**	**p**	**r**	**p**	**r**	**p**	**R**	**p**	**r**	**p**	**r**	**P**	**r**	**P**
Age	0.05	0.55	−0.10	0.44	−0.02	0.84	−0.15	0.21	−0.007	0.92	−0.27	0.02	−0.05	0.52	−0.03	0.78
Wais/hip ratio	0.09	0.24	0.10	0.41	0.05	0.52	0.13	0.27	0.06	0.44	0.08	0.51	−0.09	0.23	−0.11	0.34
Waist circumference	0.43	<0.001*	0.36	0.003	−0.08	0.27	0.07	0.57	0.05	0.48	0.08	0.49	−0.1	0.19	−0.16	0.18
Hip circumference	0.41	<0.001*	0.54	<0.001*	−0.15	0.04	0.03	0.78	0.005	0.95	0.1	0.41	−0.05	0.52	−0.11	0.36
Arm circumference	0.29	<0.001*	0.33	0.007	−0.11	0.21	−0.008	0.95	−0.05	0.47	0.04	0.72	−0.09	0.29	−0.24	0.04
BMI	0.51	<0.001*	0.49	<0.001*	−0.17	0.02	0.01	0.92	0.05	0.52	0.12	0.32	−0.06	0.42	−0.18	0.13
Glucose at 0	−0.02	0.84	0.03	0.85	0.11	0.16	−0.04	0.74	0.10	0.17	−0.20	0.09	0.04	0.58	−0. 07	0.58
Insulin at 0	0.17	0.03	0.35	0.005	0.12	0.12	0.09	0.46	0.04	0.57	0.21	0.08	−0.12	0.11	0.02	0.87
HOMA	0.13	0.11	0.31	0.02	0.15	0.06	0.04	0.78	0.05	0.51	0.11	0.36	−0.11	0.15	0.01	0.92
Triglycerides	−0.04	0.62	0.16	0.21	0.07	0.37	0.31	0.009	−0.13	0.09	−0.05	0.62	0.03	0.72	0.1	0.41
Total cholesterol	0.14	0.07	0.08	0.54	−0.01	0.87	0.1	0.42	−0.16	0.03	0.03	0.81	−0.03	0.71	0.04	0.14

*Significant p values after applying the Bonferroni correction.

Note: BMI. Body mass index.

Regarding the effect of lipodystrophy on the association between anthropometric and metabolic parameters in HIV-infected patients, a significant positive correlation was found between leptin and: insulin at 2 hours on OGTT; total, trunk, leg, arm fat evaluated by DXA; and total, SAT, VAT/SAT ratio on abdominal level evaluated by CT scan in these patients, regardless of the presence of FMR-L. Leptin was also positively associated with arm, thigh, waist, hip circumferences, BMI, glucose at 2 hours on OGTT, total cholesterol, and was negatively associated with length of cART in patients without FMR-L. Patients with FMR-L also exhibited positive associations between resistin levels and duration of HIV-infection, as well as between PAI-1 and VAT. A negative association was found between adiponectin and arm fat mass measured by DXA in FMR-L patients. A positive correlation was observed between TNF-α, fasting glucose and A1c in patients without FMR-L (Table 6).

**Table 6:** Associations between adipokines and anthropometric and metabolic parameters in patients with and without FMR defined lipodystrophy

	**Leptin**		**Resistin**		**PAI-1**		**TNF-α**		**Adiponectin**	
**Correlation between**	**Without L**	**With L**	**Without L**	**With L**	**Without L**	**With L**	**Without L**	**With L**	**Without L**	**With L**
	**n = 96**	**n = 70**	**n = 96**	**n = 74**	**n = 28**	**n = 25**	**n = 101**	**n = 73**	**n = 99**	**n = 75**
	**r**	**r**	**r**	**r**	**r**	**r**	**r**	**r**	**r**	**r**
Age	0.10	−0.01	−0.001	−0.10	−0.09	0.25	0.12	−0.19	0.04	−0.12
Duration of HIV-infection	−0.06	0.38	−0.01	0.23	0.18	−0.11	0.11	0.11	0.13	0.16
Duration of cART	−0.24	0.31	0.04	0.14	0.12	−0.16	0.05	0.05	0.04	0.21
Wais/hip ratio	0.12	0.15	0.05	0.02	0.10	0.14	0.15	−0.01	0.06	−0.19
Thigh circumf.	0.48*	−0.02	−0.17	−0.12	−0.004	0.06	−0.06	−0.02	0.07	0.07
Cervical circumf.	−0.06	−0.24	−0.02	−0.18	0.17	0.11	0.21	−0.03	−0.08	−0.08
Waist circumf.	0.53*	0.20	−0.11	0.03	−0.06	0.18	0.08	−0.01	−0.19	−0.19
Hip circumf.	0.65*	0.15	−0.20	−0.03	−0.07	0.06	−0.04	−0.04	−0.04	−0.04
Arm circumf.	0.47*	−0.06	−0.02	−0.21	−0.13	0.12	0.01	−0.21	−0.07	−0.07
CD4 cell count	−0.09	0.02	−0.01	−0.11	0.29	0.21	0.03	0.02	−0.09	−0.12
BMI	0.66*	0.17	−0.19	−0.09	−0.007	0.04	0.08	−0.02	−0.03	−0.13
Glucose at 0	0.07	−0.03	0.15	0.01	0.08	0.09	0.22	0.002	0.12	0.02
Glucose at 2 h on OGTT	0.25	0.12	0.12	0.12	−0.20	−0.12	0.06	−0.02	−0.02	−0.06
Insulin at 0	0.17	0.31	0.05	0.22	−0.10	0.03	0.09	0.12	−0.08	−0.12
Insulin at 2 h on OGTT	0.36	0.39	0.06	0.17	−0.21	−0.32	0.11	0.19	−0.08	−0.14
HOMA	0.18	0.27	0.08	0.25	−0.03	0.03	0.11	0.11	−0.06	−0.11
A1c	0.15	0.008	0.19	0.11	−0.34	−0.02	0.24	0.00	0.14	−0.02
Triglycerides	−0.004	0.03	0.06	0.17	−0.17	−0.18	−0.13	−0.17	0.08	−0.03
Total cholesterol	0.25	−0.01	−0.01	−0.06	−0.29	0.09	0.18	−0.17	−0.13	0.09
Total fat mass DXA %	0.877*	0.561*	−0.14	0.07	−0.05	−0.05	−0.07	−0.12	−0.17	−0.19
Trunk fat mass DXA %	0.858*	0.547*	−0.14	0.07	−0.05	0.001	−0.02	−0.10	−0.15	−0.20
Leg fat mass DXA %	0.825*	0.464*	−0.15	0.02	−0.06	−0.07	−0.09	−0.13	−0.15	−0.19
Arm fat mass DXA %	0.857*	0.650*	−0.12	0.08	−0.08	−0.19	−0.09	−0.10	−0.21	−0.06
Total fat mass in CT	0.774*	0.515*	−0.19	0.15	−0.07	0.07	0.13	−0.04	−0.08	−0.14
VAT in CT	0.483*	0.119	−0.18	0.14	0.05	0.41	0.05	−0.04	−0.07	−0.19
SAT in CT	0.829*	0.587*	−0.18	0.12	−0.10	−0.01	0.08	−0.06	−0.09	−0.03
_	−0.421*	−0.475*	0.10	−0.04	0.04	0.31	0.002	0.07	0.003	0.004

*p values <0.001 (significant applying the Bonferroni corretion).

The adiponectin/leptin ratio was not associated with IR [HOMA (r = −0.148, p = 0.07) or QUICKI (r = 0.148, p = 0.07)] in overall HIV patients and in male patients [HOMA (r = −0.138, p = 0.16) or QUICKI (r = 0.126, p = 0.21)]. However, a positive correlation between this ratio and QUICKI was observed [HOMA (r = −0.228, p = 0.10); QUICKI (r = 0.277, p = 0.04)] in females.

## Discussion

In acquired or genetic lipodystrophies, abnormal fat distribution *per se* is a strong determinant of IR and metabolic alterations, and the loss of fat likely precedes the metabolic complications [21-24]. Increased amounts of visceral fat accompanied by decrease in peripheral adipose tissue are associated with an increase of lipolysis and free fat acid fluxes, which together can alter adipocytokine production [25].

Regarding body fat composition, our data showed that there were differences in body fat distribution between HIV-infected patients and HIV-uninfected controls, males and females with HIV-infection and patients with or without FMR-L despite similar BMI.

Actually, HIV-infected patients had less subcutaneous adipose tissue, females had a higher fat mass and men had higher VAT and VAT/SAT ratio. FMR-L patients had less subcutaneous adipose tissue and more visceral fat mass.

### Leptin

Leptin levels were lower in HIV-infected patients namely in those with FMR-L, pointing to an association with body fat, demonstrated by the significant positive correlation between leptin and body circumferences, BMI, body fat mass evaluated by DXA and by CT scan. As previously shown, we also observed that leptin concentrations were higher in HIV-infected women compared to HIV-infected men and, therefore, the leptin sexual dimorphism remains unchanged in HIV infection [26-28]. In genetic lipodystrophy syndromes, very low plasma concentrations of leptin were observed when fat mass was profoundly reduced [29] and that correlated positively with total adiposity [30].

In our sample, HIV-infected patients with isolated central fat accumulation and mixed forms of lipodystrophy had higher BMI and leptin levels. Leptin levels were lower in those with isolated lipoatrophy, positively proportional to BMI. Most studies, that did not differentiated between patients with predominant lipoatrophy, lipohypertrophy, and mixed patterns of fat redistribution, have failed to find significantly lower leptin levels in subjects with HIV lipodystrophy [24,31,32], but when subjects with distinctive lipoatrophy have been studied separately, lower leptin levels in this subgroup of HIV lipodystrophy patients has been observed. Also, significant associations between leptin and subcutaneous fat has been reported and decreased subcutaneous fat correlated with reduced leptin levels after controlling for visceral fat quantity [33]. Total and regional adiposity were strong and positively associated with leptin in healthy controls and in HIV-infected patients, both in men and women, suggesting that adiposity remains the major determinant of plasma leptin levels in HIV infection [30]. In a prospective study, there was a significant positive correlation between the appearance of lipoatrophy and the leptin levels [32,34].

In our sample, leptin was positive and significantly associated with fasting and 2 hours insulin on OGTT and HOMA in patients with FMR-L, and glucose and insulin at 2 hours on OGTT in patients without FMR-L. The role of leptin in the modulation of insulin sensitivity is complex. Extremely low levels of leptin in congenital generalized lipoatrophies were associated with severe IR, and significantly improved with replacement of leptin in mice and humans [35]. In conclusion, leptin levels appear to be primarily determined by total adiposity in HIV-infected individuals, independently of the presence of lipodystrophy [30].

### Adiponectin

In our sample, adiponectin levels were higher in controls than in HIV-infected patients. Patients with no lipodystrophy had higher levels of adiponectin and patients with isolated central fat accumulation had the lowest levels of adiponectin. Contrary to other authors, who found higher adiponectin levels in HIV-infected women [26,27], no such gender difference was observed in our patients, and no difference regarding the presence of FMR-L was also found. We found a significant negative correlation between adiponectin and total and trunk fat mass evaluated by DXA in the HIV-infected patients, and with arm fat mass in patients without FMR-L.

Kosmiski et al., observed that both total and regional adiposity were significant and negatively correlated with adiponectin levels in a control group, similar to other reports of general populations [30]. Also, these HIV-infected patients with leg SAT below the 10th percentile of controls had significantly lower adiponectin levels than controls with similar leg SAT volumes [30]. In some studies, no association between adiponectin and total adiposity was found in HIV-infected patients, while adiponectin was positively correlated with both extremity and abdominal SAT [36,37]. These relationships suggest that the remaining SAT in HIV-infected patients with moderate to severe lipoatrophy is dysfunctional rather than simply reduced in size, as for example, patients suffering from anorexia nervosa, a condition characterized by low body weight and severe global fat depletion (although not isolated subcutaneous adipose tissue), adiponectin levels are higher [38-40]. Findings of abnormal function or loss of adipose tissue in partial lipodystrophy syndromes (due to lamin A/C or to PPAR-γ receptor mutations) and low adiponectinemia also support the hypothesis that low adiponectin concentrations in HIV-infected subjects with low levels of SAT are due to lipodystrophy *per se*[39,40]*.* Thus, lipodystrophies are characterized not only by a fat decreased but also by dysfunction or loss of adipocytes [30]. In HIV-infected men and women, with or without lipodystrophy, the normal negative relationship between visceral adiposity and adiponectin was maintained [30,36,37,41].

In HIV-infected patients, adiponectin levels correlated significantly with the percentage of limbs fat and insulin sensitivity [24,42], although some authors state that adiponectin predicts insulin sensitivity independently of the amount and repartition of body fat [28], no such association was found between adiponectin and markers of IR in our sample.

It has been reported that in HIV lipodystrophic patients, adiponectin levels were significantly lower in patients with fat redistribution (and correlated inversely with serum triglycerides). The lowest levels were in peripheral lipoatrophic and central lipohypertrophic patients playing a central role in glucose, lipid metabolism and IR in these patients [28,37,43,44].

### Ratio adiponectin/leptin

Adiponectin/leptin ratio is considered to be a better predictor of insulin sensitivity and cardiovascular risk, and is usually considered independent of body mass. Adiponectin/leptin ratio has been negatively associated with IR, metabolic defects and cardiovascular risk markers [28]. We also found a positive correlation between this ratio and QUICKI index in women. This ratio was higher in patients with no lipodystrophy and lower in patients with isolated central fat accumulation, followed by the patients with mixed forms of lipodystrophy.

### Resistin

Elevated resistin levels have been observed in obese and diabetic subjects, and increased resistin has been associated with IR in lean and obese subjects [45]. However, other studies found no association between circulating resistin levels and IR [46,47].

In our data, resistin was higher in controls and no significant differences were found in the four categories of body composition, or according to gender and presence of FMR-L in HIV-infected patients, similar to previously published data [48]. However, others found that resistin plasma levels were higher in lipodystrophic patients, being highest in those with isolated lipoatrophy [49]. Resistin was positively associated with duration of HIV-infection and with HOMA in patients with FMR-L, and negatively with hip circumference. Some authors previously demonstrated a correlation between resistin plasma levels and insulin resistance in lipodystrophic HIV patients [50] and others in HIV patients either with or without lipodystrophy [49].

### TNF- α

HIV lipodystrophy, familial partial lipodystrophy and obesity have been associated with high circulating levels of TNF-α [39,51,52]. In our sample, no significant differences in TNF-α levels were observed between controls and HIV-infected patients, in patients with or without FMR-L or regarding the four categories of fat composition. However, we found a positive correlation between TNF-α and fasting glucose and A1c in patients without FMR-L.

TNF-α homeostasis is profoundly altered by HIV-1 infection itself [53] and it has been shown that HIV-1 infected patients have elevated pre-treatment levels of TNF-α but these levels dramatically decrease when cART is initiated [54]. In our sample, patients have been treated for a long period of time (maximum 20 years) and this could potentially explain similar levels in controls and HIV-infected patients. However, other authors showed that concentrations of the components of TNF-α system are not normalized when cART is effective. We did not observe any correlation between TNF-α and adiponectin levels (r = 0.145; p = 0.06) although that has previously been described [54].

### PAI-1

Patients with no lipodystrophy and mixed forms of lipodystrophy had higher levels of PAI-1, placing them at substantial risk of cardiovascular disease via impaired fibrinolysis (54). In our sample, the lowest levels of PAI-1 were found in those with isolated central fat accumulation. No differences in PAI-1 levels between patients with or without FMR-L and according to gender were found, but a significant positive association between PAI-1 and VAT was observed in patients with FMR-L.

PAI-1 concentrations are increased in conditions characterized by increased VAT or SAT, hypertriglyceridemia, and hyperinsulinemia. Both VAT and SAT produce PAI-1, but data are controversial as to whether VAT produces more, similar, or less amounts of PAI-1 than SAT. Also, it has been suggested that PAI-1 originates from stromal cells in adipose tissue rather than from adipocytes. Patients with cART-associated lipodystrophy had elevated plasma PAI-1 concentrations [55]. HIV infected patients with fat redistribution had significant elevations in PAI-1, being waist-to-hip ratio the only significant predictor of PAI-1 [56].

Contrary to our data, He et al., found significantly increased plasma levels of PAI-1 in patients with lipodystrophy. They also found a positive correlation between plasma PAI-1 level and BMI, waist circumference, VAT, plus an inverse correlation with the percentage of limb fat. In a stepwise multiple linear regression model, VAT turned out to be the main predictor of plasma PAI-1 [57].

### Ghrelin

Ghrelin was higher in HIV-infected patients than in controls. Fasting levels of serum ghrelin has been negatively correlated with BMI, and it is decreased in obesity and increased in cachexia [58]. Contrary to our data, lower levels of ghrelin have been reported in HIV-infected lipodystrophic patients [5,59]. Falasca et al., observed higher levels of ghrelin in patients with hypertriglyceridemia, as well as a positive correlation between ghrelin and triglyceride levels in patients with hypertriglyceridemia. No such association between ghrelin and triglyceride levels were observed in our sample (r = 0.064, p = 0.42). Falasca concluded that adipokines may have crucial roles in the pathogenesis of hypertriglyceridemia and, thus, monitoring of ghrelin levels could be important to identify subjects with high cardiovascular risk [58].

### Limitations

Several limitations of this study should be noted. A cross-sectional design impairs establishing causality between cART use, lipoatrophy, and alterations in adipokine levels. It is not possible to determine whether adipokine abnormalities are due to reduced hormone expression in adipose tissue, decreased differentiation or decreased adipose cell numbers. The body fat redistribution was assessed clinically and patients divided in 4 groups but this does not preclude that some patients categorized into one subset could in fact have minor subclinical changes in their fat distribution not detectable by physical examination.

### Some aspects of the current study should be highlighted

The study was performed in a highly experienced unit in the assessment of metabolic and body fat abnormalities in HIV-infected patients; the clinical assessment of lipodystrophy was performed by the same investigator (PF); and objective definitions of lipodystrophy (Fat Mass Ratio by DXA), visceral and subcutaneous fat mass by CT were used.

## Conclusion

Leptin levels were lower in patients with lipodystrophy, indicating its close relationship with total body fat. A significant positive correlation between leptin and HOMA in patients with lipodystrophy demonstrated the role of this adipokine in insulin resistance. Adiponectin was lower in patients with isolated central fat accumulation and negatively correlated with visceral adipose tissue. The adiponectin/leptin ratio was lower in patients with isolated central fat accumulation. Patients with mixed forms of lipodystrophy had higher levels of PAI-1. Resistin was positively associated with HOMA in patients with FMR-defined lipodystrophy. Patients with no lipodystrophy and mixed forms of lipodystrophy had higher levels of PAI-1 and the lowest levels of PAI-1 were found in those with isolated central fat accumulation. Ghrelin was higher in HIV-infected patients than in controls despite the two groups being BMI-matched. Our results add to the evidence on the complexity of the relationship between adipokines, hormones related to body composition, and insulin resistance in the HIV redistribution syndrome, with different degrees of peripheral fat atrophy and central fat accumulation.

## Competing interest

All the authors declare that they have no competing interests.

## Authors’ contributions

PF conceived the study, participated in its design, in the acquisition of data and drafted the manuscript; DC conceived the study, participated in its design and drafted the manuscript; ACS performed the statistical analysis and revised critically the manuscript; AJM performed the CT scan and reviewed the data; EM critically revised the manuscript; JP performed the DXA and reviewed the data; AS critically revised the manuscript and JLM revised the study design. All authors read and approved the final manuscript.

## Pre-publication history

The pre-publication history for this paper can be accessed here:

http://www.biomedcentral.com/1471-2334/14/347/prepub
